# Low Utilization of Partograph and Its Associated Factors among Obstetric Care Providers in Governmental Health Facilities at West Shoa Zone, Central Ethiopia

**DOI:** 10.1155/2020/3738673

**Published:** 2020-07-17

**Authors:** Kefena Etita Bedada, Tufa Kolola Huluka, Gizachew Abdissa Bulto, EphremYohannesRoga

**Affiliations:** ^1^Ambo Town Health Office, Ambo, Ethiopia; ^2^Department of Public Health, College of Medicine and Health Silences, Ambo University, Ambo, Ethiopia; ^3^Department of Midwifery, College of Medicine and Health Sciences, Ambo University, Ambo, Ethiopia

## Abstract

**Background:**

Globally, prolonged and obstructed labors were among the common causes of maternal morbidity and mortality in low- and middle-income countries including Ethiopia. The World Health Organization (WHO) recommends the routine use of partograph as a key intervention to avoid prolonged and obstructed labor. Despite the recommendation, studies indicated that the partograph utilization among obstetric care providers (OCPs) is still low. Therefore, this study is aimed at assessing the level of utilization of partograph and associated factors among obstetric care providers working at health facilities in the West Shoa Zone, Central Ethiopia 2019.

**Methods:**

Facility-based cross-sectional study was conducted from February 1^st^ to 22^nd^ March 2019. A computer-generated simple random sampling technique was used to select 325 study subjects. Data were collected using a self-administered structured questionnaire and using an observational checklist. Additionally, 200 partograph charts were reviewed. Both bivariate and multivariable logistic regression analyses were used to determine the association.

**Results:**

A total of 322 obstetric care providers were included in the study, giving a response rate of 99.1%. The level of partograph utilization in the study area was revealed to be 31.1% (95% CI: 25.97-36.13). Only 3% of the reviewed partograph was recorded according to the recommended standard. In this study attending training (AOR = 3.94, 95% CI: 1.99-7.78), availability of partograph (AOR = 5.23, 95% CI: 1.69-16.22), perceived as not time-consuming task (AOR = 3.61, 95% CI: 1.19-10.96), adequate number of OCPs available (AOR = 2.92, 95% CI: 1.16-7.33), presence of supervision (AOR = 4.35, 95% CI: 2.11-8.97), having a positive attitude (AOR = 2.48, 95% CI: 1.23-5.02), availability of standard protocol in a health facility (AOR = 4.71, 95% CI: 2.31-9.60), and lack of commitment (AOR = 0.32, 95% CI: 0.16-0.63) were factors significantly associated with partograph utilization. *Conclusion and Recommendation*. Partograph utilization in the study area was found to be low. Almost all reviewed partograph charts were not recorded as to the recommended standard. Attending training, availability of partograph, perceived as it is not time-consuming, the available number of OCPs, presence of supervision, having a positive attitude, available standard protocol, and commitment were factors associated with partograph utilization. Therefore, all concerned stakeholders should emphatically consider those identified factors for intervention.

## 1. Introduction

Partograph is a simple, low-cost monitoring tool for intrapartum care recommended by the World Health Organization (WHO) which has the potential to identify obstetric complications by graphically representing the vital issues of labor progression [1]. It is the best instrument to help you detect whether labor is progressing normally or abnormally and to warn you as soon as possible if there are signs of fetal distress or if the mother's vital signs deviate from the normal range. It also causes an impact on bettering the quality of intrapartum care, maternal health, and birth outcomes [2–4].

The World Health Organization advocated the general usage of the partograph during labor, and routine use of partograph is helpful to make better decisions for the diagnosis and management of prolonged and obstructed labor [3, 5]. Partograph is one of the most potent and cost-effective tools to prevent unnecessary delays and services as the frontrunner for obstetric care givers [4, 5]. Woman's lifetime risk of dying from preventable or treatable complications of pregnancy and childbirth in sub-Saharan Africa was higher compared to that in developed regions [6–8]. Although prolonged and obstructed labors in the resource-poor settings are among the major causes of deaths, they can be prevented with the proper use of partograph [3, 8–10].

Ground from the developing countries, including Ethiopia, demonstrated that the use of partograph is poor despite preparing the tool that is simple and inexpensive for intrapartum monitoring of labor [11–14]. In Ethiopia, the major sources of maternal and neonatal morbidity and death rate are related to poor labor and delivery care. However, eighty-five percent of deaths can be prevented with cost-effective interventions like using partograph during labor and delivery [12, 15]. Skilled management of labor using a partograph, a simple chart for recording information about the advancement of labor and the status of a woman and her baby during labor, is a key to the appropriate prevention and treatment of prolonged labor and its complications [16].

Globally, about 295,000 women died during and following pregnancy and childbirth in 2017. Majority of these deaths (94%) occurred in low-resource settings, and most could have been prevented [17]. Ethiopian demographic and health survey 2016 showed that the maternal mortality ratio was 471 deaths per 100,000 live births [15]. Moreover, from the study done in Jimma University specialized hospital, the incidence of obstructed labor was 12.2% of which about 45.1% developed uterine rupture and 39.3% had sepsis with other complications [18]. These complications were preventable if correctly followed with partograph [3, 10].

The use of the partograph reduced the incidence of prolonged labor from 6.4% to 3.4%, the proportion of labor requiring augmentation from 9.9% to 8.3%, and intrapartum stillbirth rate from 0.5% to 0.3% [19]. The partograph is a very useful graphical record of the course of labor that yields optimum results when employed in labor management by obstetric care providers. Evidence abounds that the accomplishment of knowledge of its usage and ensuring proper application of that knowledge would culminate in a noteworthy decrease in the incidence and outcomes of prolonged and obstructed labor, which is reported to be associated with 8%–10% of maternal deaths [19–21].

Even though a partograph is an instrument that is helpful to manage obstructed labor and prevent prolonged labor with its ramifications, the level of usage and factors affecting utilization of partograph among obstetric care providers was not yet studied in the study area. Thus, the purpose of this study is to evaluate the extent of usage of the partograph and associated factors that inhibit obstetric care providers from consistent use of partograph in West Shoa Zone public health facilities.

## 2. Methods

### 2.1. Study Area and Period

The study was conducted from February 1^st^ to March 22^nd^, 2019, in West Shoa Zone, Oromia region, Ethiopia. The West Shoa Zone is one of the twenty zones in the Oromia region which contains 22 districts. Ambo is the city of the zone, which is located at 114 km from Addis Ababa. Concerning health facilities, there were 4 primary hospitals, 3 general hospitals, 1 referral hospital, and 92 health centers providing labor and delivery services for over 2.5 million peoples in the West Shoa Zone. According to the data from the West Shoa Zone health office, 1376 obstetric care providers were providing services at those 100 health institutions [22].

### 2.2. Study Design

The institutional-based cross-sectional study design was conducted to assess the level of partograph utilization and associated factors among obstetric care providers in the governmental health facilities of the West Shoa Zone.

### 2.3. Source and Study Populations

The source population includes all obstetric care providers (midwives, nurses, Integrated Emergency Surgery Officers, doctors, and health officers) working in all health facilities in the West Shoa Zone. A retrospective document review of all partograph from a recent delivery attended in the last three months at public health facilities from November1^st^, 2018, to January 30^th^, 2019. Study populations were all randomly selected obstetric care providers (midwives, nurses, Integrated Emergency Surgery Officers, doctors, and health officers) who have been working in all selected health facilities in West Shoa Zone. Obstetric care providers who were on leave (annual, sick, and maternity leave), who worked less than six-month duration during the data collection period were excluded from the study.

### 2.4. Sample Size Determination and Sampling Procedure

The sample size was calculated by using single-population proportion formula with the assumptions of proportion (*p*) of partograph utilization 57.3% taken from a study done in Addis Ababa [23], with 95% confidence level of *Z*Z*α*/2 = 1.96; 5% of absolute precision gives a large sample size (no. 376) to accommodate all objectives. Since the source population was less than 10,000, the correction factor was used to estimate the final required sample size which is 325 by considering 10% of the nonresponse rate.

By using the simple random sampling method, four hospitals and forty-six health centers were selected. The lists of all obstetric care providers in each health facility were considered a sampling frame. The sample was proportionally allocated to the selected health institutions according to the number of obstetric care providers. A total of 200 client's chart was selected by simple random sampling technique from four hospitals and 46 health centers.

### 2.5. Operational Definition

#### 2.5.1. Partograph Utilization

Partograph utilization refers to plotting or recording the partograph correctly and interpreting to make appropriate decisions and intervene where necessary. To assess the level of partograph utilization, two steps had been undertaken: the first footprint was asking the participants whether they were using partograph or not (with yes or no doubts). For the second measure, the respondents who replied “yes” in the first step were required to answer “how often” they accept using partograph (occasionally, sometimes, and routinely). Finally, obstetric care providers who were utilizing partograph “routinely” to monitor laboring mothers were considered utilized partograph and those who have been using sometimes and occasionally to monitor labor were considered not utilized [21, 23, 24].

#### 2.5.2. Attitude Scale

There were [10] attitude determining questions used. The responses (5: strongly agree, 4: agree, 3: uncertain, 2: disagree, and 1: strongly disagree) were scored. Providers' attitude towards partograph utilization was assessed by using a 5-point Likert scale as individuals responding strongly agree for positive attitude were given scores of 5 and 1 for those who responded as strongly disagree, while the above scores were turned back for negative attitude questions. In the end, the entire score was dichotomized into favorable and unfavorable attitudes taking the mean score as a cutoff point (mean score or more was considered having favorable attitude and less than the mean score as unfavorable attitude) [25].

#### 2.5.3. Knowledge Scale

There are 15 knowledge-assessing questions used. Correct responses were scored 1 point and incorrect 0. The scores of the items are summed up, and the total was divided by the number of the items, giving a mean score. The obstetric care providers' knowledge was considered good if it is greater than mean score and poor if it is less than mean score [23, 25].

### 2.6. Data Collection Tools and Procedure

Data were collected using a self-administered structured questionnaire and observational checklist. The questionnaire has different subsections, namely, sociodemographic, training status, knowledge, attitude towards partograph, and utilization of partograph that were used [13, 26]. Five degree holder midwives facilitated the data collection process, and three Integrated Emergency Surgery Officers (IESO) were used as supervisors. Data about the completeness of the WHO partograph was collected by using structured observational checklist by a principal investigator from each selected hospital. The constructed checklists were from the WHO-approved partograph and contain the details of items for administrative data, fetal condition, the progress of labor, drug administration, maternal condition, the time interval of recording the data, time of stopping partograph, and time of delivery.

### 2.7. Data Quality Control

The training was given for the data collectors and field supervisors for two days before the actual data collection regarding the aim of the study, the data collection tool, and procedures. For data quality assurance, one week before the actual data collection, the questionnaire was pretested on 5% of the total sample size at Holeta town health institutions. The training was focused on the art of interviewing and clarifying questions that would be unclear to answer. They also thoroughly looked into and understood the observation checklists before use. Close supervision was carried out by the investigators day to day during data collection, completeness of data had been checked daily, and corrections were made on the spot. Every day, collected data was reviewed and checked for completeness and consistency of the response.

### 2.8. Data Processing and Analysis

The collected data were checked visually for completeness, coded and entered into EPI Info version 3.5.1 statistical software, and exported into SPSS version 23 for further analysis. Descriptive summary statistics were done. Both bivariate and multivariable logistic regression analyses were used to determine the association of each independent variable with the dependent variable. Variables with a *P* value < 0.25 in the bivariate analysis were entered into a multivariable logistic regression model to adjust for the confounders. Odds ratio with 95% confidence intervals was computed to identify the presence of an association, and statistical significance was declared if *P* < 0.05. The findings of the observational assessment were analyzed using descriptive statistics. The outcomes were presented with texts, tables, and summary statistics.

### 2.9. Ethical Considerations

Ethical clearance was obtained from the Research Review and Ethics Committee of the College of Medicine and Health Sciences, Ambo University. An official letter of cooperation was given to the administrative offices of the selected health facilities. Written informed consent was obtained from each study participant. The purpose of the study was explained, and confidentiality was maintained.

## 3. Result

### 3.1. Sociodemographic Characteristics of the Study Participants

A total of 322 obstetric care providers were included in the study making a response rate of 99.1%. About 203 (63%) obstetric care providers were females and 226 (70.2%) of them were working at the health center. The mean age of the respondents was 30.87 years with a standard deviation of 4.92, and the majority of the respondents were in the age range of 25-29 years (147, 45.7%). Regarding their profession, 151 (46.9%) were midwives (diploma and degree) followed by nurse (diploma and degree) 103 (32%). One hundred ninety-six (60.9%) had a degree and above educational levels, and their mean duration of practice was 6.48 years with an SD of 4.78 (Table 1).

### 3.2. Knowledge and Attitude of Obstetric Care Providers on Partograph Utilization

**Figure 1:**
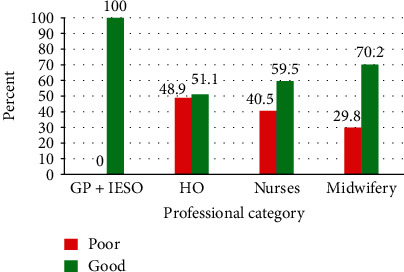
Level of knowledge on partograph among OCPs in governmental health facilities in West Shoa Zone, Central Ethiopia, 2019.

Most (259, 80.4%) of the respondents defined the partograph correctly. Most of the respondents (267, 82.9%) used partograph before and 208 (64.6%) know the components of partograph. Similarly, 271 (84.2%) respondents knew alert and action lines (Table 2). One hundred eighty-five (57.5%) (95% CI, 52.02-62.9) respondents had good overall knowledge, while 137 (42.5%) of them had poor knowledge of partograph (Figure 1).

**Figure 2:**
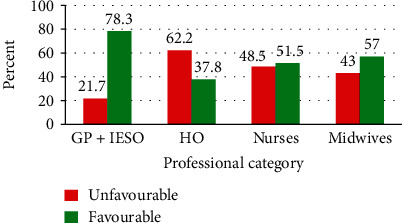
Attitude towards partograph utilization among obstetric care providers in governmental health facilities in the West Shoa Zone, Central Ethiopia, 2019.

More than half of the obstetric care providers (54%) (95% CI 48.6-59.5) generally had a favorable/positive attitude toward partograph (Table 3). Medical doctors and IESO (78.3%) and midwives (57.0%) have a more favorable attitude than others (Figure 2).

### 3.3. Partograph Utilization

Most of the respondents said that they had used partograph (197, 61.2%) to monitor the condition of laboring mothers, most of the respondents (252, 78.3%) reported that the partograph is always available in their health facility, and 297 (92.2%) said that partograph is a mandatory tool to improve the quality of care given to the laboring woman. One hundred sixty-eight (52.2%) have no policy or standard protocol for guidance on the use of partograph. Frequency of partograph utilization by obstetric care providers indicates that 100 (31.1%) of them use it routinely, 106 (32.9%) use partograph sometimes, 73 (22.7%) use partograph occasionally, and 43 (13.4%) do not use partograph. However, less than half (39, 40.6%) of the obstetric care providers from hospitals have used partograph and 61 (27%) respondents were using it in health centers.

Among medical records reviewed, only about 173 (86.5%) records have partograph forms while 7 (3.5%) used plain paper and 20 (10%) used patient history sheets to follow laboring mothers. The pattern of monitoring and recording from components of partograph indicated that only 6 (3%) partograph was recorded as to the WHO's standard (Table 4).

Majority (313, 97.2%) of the respondents said that they learned about partograph during a stay at a university or college. Most of the respondents (197, 61.2%) had never attended training while only 125 (38.8%) had attended a workshop/training on basic or comprehensive emergency obstetric and newborn care. Majority (113, 90.4%) of them have taken the training or orientation a year ago.

### 3.4. Reasons for Not Using Partograph among Obstetric Care Providers

The reason reported by most of the study participants for not routinely using partograph were partograph chart not available, not trained about partograph, shortage of health care personnel, and the absence of obligation from hospital/health center policy to perform (Table 5). The main reasons mentioned by participants who were aware of the partograph, but not using the partograph for monitoring women in labor were as follows: lack of orientation/training on how to use (112, 34.8%), availability of other methods of observation (43, 13.4%), lack of commitment (128, 39.8%), workload (199, 61.8%), and lack of supervision (172, 53.4%).

### 3.5. Factors Associated with Partograph Utilization among OCPs

In the bivariate analysis, the factors found to be significantly associated with partograph utilization and all candidate variables were analyzed by a multivariable logistic regression model.

Multiple logistic regression analysis indicated that obstetric care providers having a policy or standard protocol for guidance on the use of partograph were almost 5 times more likely to utilize the partograph than those who did not have in their health facility (AOR = 4.71, 95% CI: 2.31, 9.60). Those obstetric care providers who received training on partograph were almost 4 times more likely to use partograph than those who did not (AOR = 3.94, 95% CI: 1.99, 7.78). Those who had a partograph chart always available were 5 times more likely to use partograph than those where partograph is not always available (AOR = 5.23, 95% CI: 1.69, 16.22), and those obstetric care providers who have a favorable attitude toward partograph were two times more likely to utilize partograph than their counterparts (AOR = 2.48, 95% CI: 1.23, 5.02). Furthermore, those respondents that had more health care personnel staff in their health facility were almost three times more likely to utilize partograph than a shortage of health care personnel staff in their health facility (AOR = 2.48, 95% CI: 1.23, 5.02). Likewise, obstetric care providers who reported lack of commitment about the use partograph were 68% less likely to utilize the partograph for monitoring mothers in labor compared to those who were committed to utilizing the partograph (AOR = 0.32, 95% CI: 0.16, 0.63) (Table 6).

## 4. Discussion

The level of partograph utilization in the study area was revealed to be 31.1% (95% CI: 25.97-36.13). The current finding is in line with the findings from Asella referral and teaching hospitals (26%) [27], the Amhara region (29.3%) [13], Uganda (30%) [26], Nigeria (32.4%) [28], and Port Said and Ismailia cities (35.9%) [25].

But the current finding was higher than the findings from Jimma Referral Hospital Ethiopia (6.3%) [29], urban hospitals in Lilongwe-Malawi (3.9%) [30], and Mulanje District Hospital in Malawi (10%) [31]. The higher prevalence rate in this study is attributed to the availability of partograph, having a policy or standard protocol, better supervision, and positive attitudes towards its use.

This finding is lower than that of the studies done in Addis Ababa (57.4%) [23], Western Oromia hospitals (89.1%) [32], East Gojam Zone (53.85%) [14], Cameroon (58.2%) [33], and the Niger Delta of Nigeria (98.8%) [34]. These differences might be due to the differences in the place of the study that may be explained with different strategies in partograph implementation, selected facility type, data collection methods, sample size, different levels of knowledge, and attitudes of care providers towards partograph utilization.

Attending training or workshop of OCPs was more likely significantly associated with the utilization of the partograph. This finding is consistent with previous studies done in the Amhara region [13], Tanzania [25], and in North Shoa [11] and Eastern Gojam Zone of Ethiopia [14] which is more likely associated with OCPs interviewed that had been previously trained in-service to use the partograph. However, studies done in Western Oromia hospitals indicated less likely utilization than counterparts [32]. This finding points to the need that OCPs should get periodic on-the-job refresher training on the obstetric care that has been overlooked and better make use of the partograph with post-follow-up of training, and this may be possibly explained by the fact that training would improve the status of knowledge about the area of interest.

Those participants who were reporting plotting of the partograph was not an additional time-consuming task were more likely to utilize partograph than those who reported plotting partograph was an additional time-consuming task. This study is not in line with a study done in Nigeria [34] and Addis Ababa [23]. Additionally, studies conducted in Nigeria in the Enugu Metropolis and Southwest Region of Cameroon showed that some midwives often think that completing the partograph is an additional time-consuming task and as such have no understanding of how it can save a woman's life [35, 36]. The probable reason for this discrepancy might be a shortage of staff and consequent workload and may be also ignorance of the importance/benefit of the partograph.

Obstetric care providers having a policy or standard protocol for guidance on the use of partograph in their health facility are more likely to use partograph than counterparts. This implies that it might be that a shortage of health care personnel brings workload which means that where there was a shortage of health care personnel; appropriate actions may vary depending on the setting: augmentation of labor, operative delivery, or just timely referral to a higher level of care. Standard management protocols on the actions to be taken based on partograph that is available for use at first and referral system and should be used to help in decision-making. Obstetric care providers were not likely to use partograph which leads to poor management of laboring women [10, 20, 32].

Those OCPs who were supervised have more likely utilized partograph than the counterpart; for effective utilization of partograph and partograph-based decision-making, OCP's knowledge advancement through refresher training, including practical demonstration, supportive supervision, and on-site partograph audits by trained supervisors, should also be prioritized [26, 36, 37]. The possible reason could be due to the availability of well-designed and coordinated programs like the strength of mentorship and supportive supervision of OCPs. Other reasons might be the competency level and background characteristics of the study participants. Strengthening the integrated supportive supervision with in-services would further enable the workers to implement their decisions in case of an obstetric emergency.

The availability of the partograph was a big hindrance to its utilization. Similarly, a study in Rujumbura Health Subdistrict in Southwestern Uganda found that the various health centers had sufficient numbers of the partograph although their utilization was low [26]. However, other sites have documented unavailability as a problem, for example, in Central Ethiopia, Nigeria, and South Africa [10, 23, 28, 34]. In our study, the unavailability of partograph may be due to the low preprinted material of partograph by the health institutions and/or the adequacy of the health facility's stationery supply system and other required essential health facility supplies such as blood pressure cuffs and urine test strips that also had gaps not always available.

Having adequate health care personnel staff had three times more likely to utilize partograph than counterparts. The employment of adequate staff was reported by the respondents as a factor that could facilitate their use of the partograph. This is because the increase in the number of workers will bring about a decrease in work overload and thus their provision of quality care. The finding is supported by the studies in Enugu Metropolis and Cambodia which found that more staff on duty was associated with high utilization of the partograph and if there was more partograph completed, there were more staff on duty. This finding is also in line with the finding of Saviola, Arez, Raddi, Sudha, and Metgu in South Asia [38], who inferred that the planned teaching program was effective to improve knowledge and skills on partograph.

The result of this study also revealed the existence of a significant association between participants' attitudes towards the partograph and utilization of it. This is in agreement with the studies done in Nigeria [34] and Ethiopia in Addis Ababa, Oromia, and the Amhara region [11, 13, 23]. This could be because having a good attitude towards partograph utilization might come after having knowledge about partograph that may influence the utilization of partograph.

### 4.1. Strength and Limitation of the Study

The study was conducted at 50 health facilities found in the West Shoa Zone, which covers half of those health facilities providing the service for over 2.5 million peoples. The limitations of this study could include the fact that there might be social desirability bias which may cause the obstetric care providers who took part in this study to overstate their use of the partograph. However, numerous scientific procedures were employed to minimize the possible effects of social desirability. Since the study design also uses a retrospective review of records for secondary data, some relevant data were missing due to inconsistency and incomplete documentation in the patient files. Progress notes in women's files were used to get the missing data from the partograph.

## 5. Conclusion

Partograph utilization in the study area was found to be low. Shortage of health care personnel, lack of supervision, partograph chart availability, having a favorable attitude towards partograph, and additional time-consuming tasks for the inadequate staff were factors affecting partograph utilization. Almost all reviewed partograph charts were not recorded as to the recommended standard. Therefore, continuously providing the necessary resources needed for the utilization of the partograph such as partograph charts, observation tools, gloves, blood pressure apparatus, thermometer, and urine analysis kit should have to be maintained. Furthermore, there should be regular supportive supervision by senior care providers, and they should provide on-the-job training on how to use partograph for OCPs and ensure deployment of an adequate number of OCPs based on the available caseloads.

## Figures and Tables

**Table 1:** Sociodemographic characteristics of obstetric care providers in governmental health facilities in West Shoa Zone, Central Ethiopia, 2019.

Variable (*n* = 322)		Frequency	Percent
Health facility type	Hospital	96	29.8
	Health center	226	70.2

Sex	Male	119	37.0
	Female	203	63.0

Age category in years	20-24	11	3.4
	25-29	147	45.7
	30-34	95	29.5
	35-39	41	12.7
	>40	28	8.7

Professional category	GP & IESO	23	7.1
	HO	45	14.0
	Nurse	103	32.0
	Midwives	151	46.9

Service years	≤3 years	104	32.3
	4-6 years	93	28.9
	7-9 years	53	16.5
	>9 years	72	22.4

**Table 2:** Knowledge of partograph among OCPs in governmental health facilities in West Shoa Zone, Central Ethiopia, 2019.

Variables		Frequency	Percent
Definition of partograph	Correct	259	80.4
	Incorrect	63	19.6
Fetal conditions components	Correct	207	64.3
	Incorrect	115	35.7
Progress of labor components	Correct	216	67.1
	Incorrect	106	32.9
Maternal conditions components	Correct	109	33.9
	Incorrect	213	66.1
Knows alert & action lines	Correct	271	84.2
	Incorrect	51	15.8
The function of alerting at the health center	To give early warning	148	46.0
	It indicates for augmentation	26	8.1
	It does not indicate any	3	.9
The function of alerting at the hospital	As a warning for extra vigilance	67	20.8
	Transfer laboring mothers	17	5.3
	It does not indicate any	9	2.8
The function of the action line at hospital	It indicates the critical point	80	24.8
	Followed for additional 2 hrs	13	4.0
Knows when to start plotting on partograph	Correct	233	72.4
	Incorrect	89	27.6
For whom to use partograph	Correct	284	88.2
	Incorrect	38	11.8
When did you fill partograph	After delivery of the baby	87	27.0
	While the woman is still in labor	235	73.0
Definition of prolonged labor	Correct	149	46.3
	Incorrect	173	53.7
Definition of obstructed labor	Correct	209	64.9
	Incorrect	113	35.1
Overall level of knowledge on partograph	Correct	185	57.5
	Incorrect	137	42.5

**Table 3:** Attitude towards partograph utilization among OCPs in governmental health facilities in West Shoa Zone, Central Ethiopia, 2019.

Variables	OCPs respondents (*n* = 322)	
	Unfavorable attitudes *N* (%)	Favorable attitudes *N* (%)
Partograph is a tool to monitor labor	90 (28)	232 (72)
Partograph used in all normal labor	105 (32.6)	217 (67.4)
Partograph should be used for high-risk mothers only	131 (40.7)	191 (59.3)
Use of partograph decreases risks of complication on mother and/or newborn	85 (26.4)	237 (73.6)
Partograph helps early identification of cases for transfer, augmentation, and/or surgery	87 (27)	235 (73)
Using partograph increases the quality and regularity of all observations for mother and fetus	80 (24.8)	242 (75.2)
Using partograph is an only time-consuming task without any benefit	178 (55.3)	144 (44.7)
Using partograph is the responsibility of midwife only	79 (24.5)	243 (75.5)
Using partograph is not an appropriate method of monitoring	122 (37.9)	200 (62.1)
Using partograph misleads management as the progress of labor	135 (41.9)	187 (58.1)
Total attitude	Favorable	174 (54)
	Unfavorable	148 (46)

**Table 4:** Completeness of filled partograph from reviewed records in governmental health facilities of West Shoa Zone, Central Ethiopia, 2019.

Variable	Frequency (*N* = 200)and percent		
	Recorded as standard	Substandard	Not recorded
FHB	92 (46)	91 (45.5)	17 (8.5)
Membrane & liquor	47 (23.5)	76 (38)	77 (38.5)
Molding of the fetal skull	57 (28.5)	31 (15.5)	112 (56)
Cervical dilatation	130 (65)	44 (22)	26 (13)
Descent of the fetal head	25 (12.5)	83 (41.5)	92 (46)
Uterine contraction	92 (46)	39 (19.5)	69 (34.5)
Additional medication & IV fluids	98 (49)	83 (41.5)	19 (9.5)
Maternal pulse	33 (16.5)	107 (53.5)	60 (30)
Blood pressure	56 (28)	115 (57.5)	29 (14.5)
Temperature	27 (13.5)	8 (4)	165 (82.5)
Protein, acetone & urine volume	14 (7)	59 (29.5)	127 (63.5)
Overall recording status from the reviewed partograph charts	6 (3)	138 (69)	56 (28)

**Table 5:** Reason mentioned for not using partograph among obstetric care providers in governmental health facilities in West Shoa Zone, Central Ethiopia, 2019.

Variable		Frequency	Percent
Partograph chart was not available	No	277	86.0
	Yes	45	14.0
Not trained about partograph	No	190	59.0
	Yes	132	41.0
Shortage of health care personnel	No	255	79.2
	Yes	67	20.8
Time-consuming task due to the low number of staff	No	263	81.7
	Yes	59	18.3
Its advantage not that much appreciable	No	265	82.3
	Yes	57	17.7
No managerial support and motivation for the use	No	221	68.6
	Yes	101	31.4
Absence of obligation from HFs policy to perform	No	264	82.0

**Table 6:** Factors associated with partograph utilization of bivariate and multivariable logistic regression analysis among OCPs in governmental health facilities in West Shoa Zone, Central Ethiopia, 2019.

Variable		Partograph utilization		COR (95% CI)	AOR (95% CI)
		Utilized	Nonutilized		
		No. (%)	No. (%)		
Attended training/orientation	Yes	66 (20.5)	59 (18.3)	5.36 (3.21-8.93)^∗^	3.94 (1.99-7.78)^∗^
	No	34 (10.6)	163 (50.6)	1	
Have a policy or standard protocol	Yes	69 (21.4)	85 (26.4)	3.59 (2.17-5.93)^∗^	4.71 (2.31-9.60)^∗^
	No	31 (9.6)	137 (42.6)	1	
Partograph always readily available	Yes	94 (29.2)	158 (49)	6.35 (2.65-15.22)^∗^	5.23 (1.69-16.22)^∗^
	No	6 (1.9)	64 (19.9)	1	
Attitude toward partograph utilization	Favorable.	68 (21.2)	106 (32.9)	2.33 (1.42-3.82)^∗^	2.48 (1.23-5.02)^∗^
	Unfavorable.	32 (9.9)	116 (36)	1	
Plotting partograph is a time-consuming task	No	90 (28)	173 (53.7)	2.55 (1.23-5.27)^∗^	3.61 (1.19-10.96)^∗^
	Yes	10 (3.1)	49 (15.2)	1	
Supervision	Yes	64 (19.9)	86 (26.7)	2.81 (1.72-4.59)^∗^	4.35 (2.11-8.97)^∗^
	No	36 (11.2)	136 (42.2)	1	
Commitment	No	35 (10.9)	159 (49.4)	0.21 (0.13-0.35)^∗^	0.32 (0.16-0.63)^∗^
	Yes	65 (20.2)	63 (19.6)	1	
Adequate manpower	Yes	87 (27)	168 (52.2)	2.15 (1.11-4.16)^∗^	2.92 (1.16-7.33)^∗^
	No	13 (4)	54 (16.8)	1	1

^∗^Statistically significant at *P* value < 0.05 of adjusted 95% CI; 1 = reference category.

## Data Availability

The data used to support the findings of this study are available from the corresponding author upon request.

## Abbreviations

IESO: Integrated Emergency Surgery Officer
OCP: Obstetric care providers
WHO: World Health Organization.
